# Supplementary material for: Extraocular, rod-like photoreceptors in a flatworm express xenopsin photopigment
Source: eLife. 2019 Oct 22;8:e45465. doi: 10.7554/eLife.45465 (PMC6805122; doi:10.7554/eLife.45465)
Supplement: Transparent reporting form [file elife-45465-transrepform.docx]

***eLife’s* transparent reporting form**

We encourage authors to provide detailed information *within their submission* to facilitate the interpretation and replication of experiments. Authors can upload supporting documentation to indicate the use of appropriate reporting guidelines for health-related research (see [EQUATOR Network](http://www.equator-network.org/%20)), life science research (see the [BioSharing Information Resource](https://biosharing.org/)), or the [ARRIVE guidelines](http://www.plosbiology.org/article/info:doi/10.1371/journal.pbio.1000412) for reporting work involving animal research. Where applicable, authors should refer to any relevant reporting standards documents in this form.

If you have any questions, please consult our Journal Policies and/or contact us: [editorial@elifesciences.org](mailto:editorial@elifesciences.org).

**Sample-size estimation**

- You should state whether an appropriate sample size was computed when the study was being designed
- You should state the statistical method of sample size computation and any required assumptions
- If no explicit power analysis was used, you should describe how you decided what sample (replicate) size (number) to use

Please outline where this information can be found within the submission (e.g., sections or figure legends), or explain why this information doesn’t apply to your submission:

All information about sample sizes is available in both the figure legends and/or materials and methods.

Serial SEM data of ciliary photoreceptors: due to the time and resources needed to generate these data we only analyzed one series at each thickness and magnification.

**Replicates**

- You should report how often each experiment was performed
- You should include a definition of biological versus technical replication
- The data obtained should be provided and sufficient information should be provided to indicate the number of independent biological and/or technical replicates
- If you encountered any outliers, you should describe how these were handled
- Criteria for exclusion/inclusion of data should be clearly stated
- High-throughput sequence data should be uploaded before submission, with a private link for reviewers provided (these are available from both GEO and ArrayExpress)

Please outline where this information can be found within the submission (e.g., sections or figure legends), or explain why this information doesn’t apply to your submission:

For the xenopsin cellular assays (illustrated in Fig.5), a total of 3 experiments were performed (biological replicates), with 3-4 technical replicates in each biological replicate. These details are included in the figure legend of Figure 5 and Supplementary Figure 4, and in Methods “G-protein selection of *Maritigrella* crozieri xenopsin”).

For protein and mRNA expression analysis using immunofluorescence and *in situ* hybridization, the number of specimens examined is given in the figure legends of Figures 2,3 and 4. >3 biological replicates were examined in each experiment.

**Statistical reporting**

- Statistical analysis methods should be described and justified
- Raw data should be presented in figures whenever informative to do so (typically when N per group is less than 10)
- For each experiment, you should identify the statistical tests used, exact values of N, definitions of center, methods of multiple test correction, and dispersion and precision measures (e.g., mean, median, SD, SEM, confidence intervals; and, for the major substantive results, a measure of effect size (e.g., Pearson's r, Cohen's d)
- Report exact p-values wherever possible alongside the summary statistics and 95% confidence intervals. These should be reported for all key questions and not only when the p-value is less than 0.05.

Please outline where this information can be found within the submission (e.g., sections or figure legends), or explain why this information doesn’t apply to your submission:

In the xenopsin cellular assays the biological and technical replicates (see above) all gave the same qualitative response and are sufficient to support the conclusions about Gi/o signalling as presented.

We did a 1- way ANOVA to compare the fluorescence of HEK cells expressing an opsin versus HEK cells with no opsin (Figure 6 - figure supplement 1), p-values are indicated in the figure and legend.

(For large datasets, or papers with a very large number of statistical tests, you may upload a single table file with tests, Ns, etc., with reference to sections in the manuscript.)

**Group allocation**

- Indicate how samples were allocated into experimental groups (in the case of clinical studies, please specify allocation to treatment method); if randomization was used, please also state if restricted randomization was applied
- Indicate if masking was used during group allocation, data collection and/or data analysis

Please outline where this information can be found within the submission (e.g., sections or figure legends), or explain why this information doesn’t apply to your submission:

No group allocation or masking was performed in these experiments.

**Additional data files (“source data”)**

- We encourage you to upload relevant additional data files, such as numerical data that are represented as a graph in a figure, or as a summary table
- Where provided, these should be in the most useful format, and they can be uploaded as “Source data” files linked to a main figure or table
- Include model definition files including the full list of parameters used
- Include code used for data analysis (e.g., R, MatLab)
- Avoid stating that data files are “available upon request”

Please indicate the figures or tables for which source data files have been provided:

Figure 1 – source data 1 – opsin amino acid sequences used in the opsin phylogeny in Figure 1

Figure 5 – source data 1 – G alpha subunit amino acid sequences used in the phylogeny in Figure 5 – figure supplement 1
